# Fulminant Leptospirosis (Weil's disease) in an urban setting as an overlooked cause of multiorgan failure: a case report

**DOI:** 10.1186/1752-1947-5-7

**Published:** 2011-01-14

**Authors:** Elias Maroun, Anurag Kushawaha, Elie El-Charabaty, Neville Mobarakai, Suzanne El-Sayegh

**Affiliations:** 1 Staten Island University Hospital, 475 Seaview Avenue, Staten Island, NY 10305 USA

## Abstract

**Introduction:**

Leptospirosis has recently come to international attention as a globally important re-emerging infectious disease. Our case is unusual given the season, location and setting in which leptospirosis occurred. According to the New York City Board of Health, there were only two other cases of leptospirosis in New York City in the year that our patient was diagnosed.

**Case presentation:**

A 49-year-old healthy Chinese man presented to our hospital with sepsis and multiorgan failure. The patient did not respond to antibiotics and his multiorgan failure worsened. His workup did not show any significant findings except for a positive nasopharyngeal swab result for influenza A. Later the patient developed hemoptysis with evidence of bilateral infiltrates on radiography. His status mildly improved after he was started on steroids. Eventually, a microagglutination test confirmed the presence of antibodies against *Leptospira icterohaemorrhagiae. *The patient subsequently recovered after a course of intravenous antibiotics.

**Conclusion:**

The case of fulminant leptospirosis presented here should serve to alert health care providers and the general public to the clinical importance of this severe, sometimes fatal, disease. Leptospirosis should be considered early in the diagnosis of any patient with acute, non-specific febrile illness with multiorgan system involvement or high fever in a returning traveler. In addition, not only should it be considered in tropical and rural areas between late summer to early fall, but also in any location or time if the risk factors are present.

## Introduction

Leptospirosis is a zoonosis of worldwide distribution caused by infection with *Leptospira interrogans*, a pathogenic spirochete. The most important reservoirs are rodents, predominantly rats. Urinary shedding of organisms from infected animals is the most significant source of *Leptospira *spp. The majority of patients manifest a mild, anicteric febrile illness, but a minority of patients develop a severe form with multiorgan involvement, called Weil's disease. Weil's disease is characterized by multisystem dysfunction and can present with high fever, significant jaundice, renal failure, hepatic necrosis, pulmonary involvement, cardiovascular collapse, neurologic changes and hemorrhagic diathesis.

## Case presentation

A 49 year-old man of Chinese descent with no medical history presented in mid-January to our hospital for a history of fever (102.5°F), myalgias, and severe bilateral calf pain that began six days prior.

He had a 30 pack-year history of cigarette smoking, drank four to six beers almost every day for two years and smoked marijuana occasionally. He denied recent travel, owned two healthy pets and worked as a construction worker. The patient denied any recent travel outside the USA. He denied recent antibiotic exposure or sick contacts.

Vital signs in the emergency department were notable for a temperature of 101°F, pulse of 120 beats/min and blood pressure of 156/63 mm Hg. The patient was alert and oriented. The ocular examination was notable for scleral icterus. The skin appeared jaundiced, the lungs were clear to auscultation, the abdomen was soft, bilateral lower extremity tenderness was noted and dorsal pedal pulses were present bilaterally.

Initial laboratory study results were notable for a creatinine of 2.3 mg/dL, platelets of 58,000 cells/mm^3^, hemoglobin of 12.8 G/dL, white blood cell count of 8.9 × 10^3 ^cells/mm^3 ^with lymphopenia of 2.4%, total bilirubin of 4.3 mg/dL, direct bilirubin of 2.6 mg/dL, alkaline phosphatase (ALP) of 143 U/L, aspartate aminotransferase (AST) of 201 U/L, alanine aminotransferase (ALT) of 246 U/L, and creatine kinase of 1219 U/L. The urine analysis showed moderate hematuria but no proteinuria. Results of lower extremity Doppler ultrasonography and chest radiography were negative, and electrocardiography showed normal sinus rhythm at 76 beats/min.

The patient was admitted to the intensive care unit for sepsis and multiorgan dysfunction. Intravenous (IV) ceftriaxone and vancomycin were initiated with aggressive fluid resuscitation.

The following day, the patient's acute renal failure, hyperbilirubinemia, anemia and thrombocytopenia worsened. Serologic test results for acute hepatitis A, B and C infections were negative. A peripheral smear showed no schistocytes. Levels of C3, C4, antinuclear antibodies, anti-dsDNA, antineutrophil cytoplasmic antibodies, and anti-glomerular basement membrane antibodies were within normal limits. Renal ultrasonography results were normal. Computed tomography of the abdomen showed pancolitis, cholelithiasis and nephromegaly.

On the third day, the patient had worsening oxygenation with slight hemoptysis and developed new inferior bilateral infiltrates. Physical examination revealed the development of fine crackles at the bases of his lungs. Arterial blood gas on 2 L of oxygen revealed a pH of 7.45, PCO_2 _(partial pressure of carbon dioxide) of 28, PO_2 _(partial pressure of oxygen) of 55, oxygen saturation of 90% and bicarbonate of 19.5. The antibiotic regimen was broadened to IV cefepime, and vancomycin was continued. The right upper quadrant sonogram showed thickening of the gallbladder wall. Urine legionella antigen and serum HIV antibody results were negative. Initial blood cultures and sputum culture results were negative. The patient's acute renal failure, hyperbilirubinemia, anemia and thrombocytopenia continued to deteriorate. The patient was started on 125 mg of methylprednisone every six hours for five days and desmopressin for suspicion of alveolar hemorrhage in the presence of renal failure.

On the fourth hospital day, a nasopharyngeal swab for influenza A was conducted, and results were positive. All antibiotics were stopped, and the patient received one dose of oseltamivir. The patient's clinical status and renal failure started to improve, but the cholestatic picture was worsening, with a total bilirubin of 64.7 mg/dL, direct bilirubin of 44.8 mg/dL, AST of 87 U/L and ALT of 121 U/L.

On the fifth day, the patient developed a maculopapular, nonpruritic rash on the face, torso, abdomen and upper extremities involving the palms. Biopsy was done and showed lichenoid dermatitis (drug reaction). Serum leptospira antibodies were sent to the NYC Board of Health for specialized testing. While the patient was taking corticosteroids, the hyperbilirubinemia, thrombocytopenia, hemoptysis and renal failure resolved over the next few days. The final laboratory study results were notable for a creatinine of 0.8 mg/dL, total bilirubin of 1.8 mg/dL and platelets of 165 cells/mm^3^. There was a near complete resolution of the chest radiography findings. Furthermore, the rash and the patient's calf pain gradually improved, and the patient was subsequently discharged.

Two weeks later, the NYC Board of Health reported the antibodies for leptospira as positive. A microagglutination test (MAT) confirmed the serovar *Leptospira icterohaemorrhagiae*, with the titers 1:6400. The patient was recalled for insertion of an indwelling catheter for a 14-day course of IV ceftriaxone after which he improved. Retrospectively, after having been asked about any epidemiologic factors that placed him at risk for contracting leptospirosis, the patient admitted to having been in contact with rat urine with his bare hands at a construction site in NYC.

## Discussion

Leptospirosis is a zoonosis of worldwide distribution caused by infection with *L. interrogans*, a pathogenic spirochete. The organism infects a variety of animals, especially rodents and animals associated with farming. Humans represent only incidental infection usually through work-related contact through skin or mucous membranes, typically after exposure to water or soil contaminated with urine from an infected animal or via drinking of or bathing in contaminated water. The main occupational groups at risk are farm workers, field agricultural workers, plumbers, sewer workers, sanitation workers and military troops.

Leptospira are spiral-shaped, thin, motile organisms with flagella. The most common serovars are *icterohaemorrhagiae*, which are usually found in rats (*Rattus norvegicus)*. Urinary shedding of organisms from infected animals is the most significant source of *Leptospira *spp. because the spirochetes can persist for long periods of time in the renal tubules.

The natural course of leptospirosis comprises of two distinct clinical phases: septicemic and immune. Humans typically become ill seven to 12 days after exposure to leptospires. The first stage is called the septicemic phase (leptospiremic phase) because the bacteria may be isolated from blood cultures and cerebrospinal fluid (CSF). This phase is characterized by a nonspecific flulike illness with sudden onset of high fever, headache, myalgias (classically involving the paraspinal, calf and abdominal muscles) [1] and conjunctival suffusion. Conjunctival suffusion (reddening of the eye surface) is a characteristic physical finding in leptospirosis, and its presence in a patient with a nonspecific febrile illness should raise suspicion for diagnosis.

The second stage is called the immune phase (leptospiruric phase) when circulating antibodies can be detected and the bacteria can be isolated from the urine. This stage occurs as a result of the body's immunologic response by producing immunoglobulin M antibodies and can last longer than one month. During this stage, specific organ damage can be observed. Aseptic meningitis is one of the most important clinical syndromes that can occur in 80% of patients during the immune phase. Renal symptoms, such as uremia, azotemia, pyuria and hematuria, may occur. Pulmonary manifestations, although usually benign, can be potentially life threatening and range from chest pain, cough and dyspnea to pulmonary hemorrhage or acute respiratory distress syndrome. An increase in liver enzymes (up to five times normal) with a disproportionately high total bilirubin has been described as a prognostic indicator in leptospirosis [2]. Varying degrees of jaundice, pancreatitis, hepatomegaly and myocarditis can also occur.

Weil's disease is the most severe form of leptospirosis. Patients can present with high fever (>40°C), significant jaundice, renal failure, hepatic necrosis, pulmonary involvement, cardiovascular collapse, neurologic changes and hemorrhagic diathesis, with a variable clinical course. Weil's disease can occur at the end of the first stage and peaks during the second stage but can occur at any time during acute leptospirosis as a single, progressive illness.

Acute renal failure is one of the most common complications of severe leptospirosis. Renal leptospirosis is usually described as a combination of acute tubular damage and interstitial nephritis.

A particularly serious type of lung involvement called severe pulmonary hemorrhagic syndrome is considered to be a major cause of death in patients with Weil's disease in developing countries, with profuse lung hemorrhage dominating the clinical picture [3].

Hepatic dysfunction is usually mild and reversible. Liver dysfunction in severe leptospirosis can be seen as conjugated serum bilirubin levels may increase to above 80 mg/dL, accompanied by modest elevations in transaminases, which rarely exceed 200 U/L [4].

Variable degrees of thrombocytopenia have been reported with leptospirosis. The pathogenesis of thrombocytopenia and hemorrhagic diathesis in leptospirosis is not well understood.

Overall, Weil's syndrome has a mortality rate of 5% to 10%. Important causes of death include renal failure, cardiopulmonary failure and widespread hemorrhage [5].

The diagnosis of leptospirosis requires a high degree of clinical suspicion because the disease's numerous manifestations can mimic other tropical infections or other nonspecific febrile illnesses, as well as noninfectious diseases such as small vessel vasculitides, systemic lupus erythematosus or even malignancies. The initial diagnosis of leptospirosis remains a clinical one, a presumed analysis in the appropriate epidemiologic and clinical context. Routine laboratory testing is nondiagnostic but may show elevated erythrocyte sedimentation rate, peripheral leukocytosis, variable degrees of cytopenias, mildly increased aminotransferases and increased serum bilirubin and ALP.

Isolation of the organism by culture of clinical specimens (blood, CSF, urine) during the first seven to 10 days of the illness is considered the gold standard of diagnosis. However, this method is difficult, requires longer than 16 weeks because initial growth may be slow and has a low sensitivity and specificity. The majority of leptospirosis cases are diagnosed by serologic testing of which MAT is most common

The vast majority of infections with leptospira are self-limiting, and it remains controversial if antimicrobials produce benefit in cases of mild leptospirosis without end-organ damage. The current choices of treatment for mild leptospirosis include oral doxycycline and amoxicillin. Parenteral high-dose penicillin G has long been considered the treatment of choice of fulminant leptospirosis. Recent trials have demonstrated that the broad-spectrum third generation cephalosporins cefotaxime and ceftriaxone are also acceptable agents for patients with severe leptospirosis [6,7].

The use of steroids in patients with leptospirosis has not been well established. In the current case, the improvement of the patient's renal dysfunction, thrombocytopenia and hemoptysis may be attributed to the introduction of steroids. Several case reports have described the beneficial effects of glucocorticoids in severe leptospirosis with pulmonary hemorrhage [8], thrombocytopenia [9] and renal failure [10,11].

Public health measures to prevent and reduce leptospirosis include identification of contaminated water sources, rodent control, prohibition of swimming in waters where risk of infection is high and informing persons of the risk involved in recreational water activities.

In the case of our patient, the diagnosis of leptospirosis was not initially considered because potential risk factors were not identified at the outset. The majority of cases of leptospirosis occur in the tropics, with infrequent incidences in temperate regions. Adding another atypical facet to the patient's presentation, in the United States, the majority of cases occur in the Southern and Pacific coastal states, with Hawaii having the most reported cases. Also, our patient presented in the wintertime. Most cases of leptospirosis occurring in temperate areas occur in the late summer to early fall [1]. According to the NYC Board of Health, between 2008 and summer 2009, there were only three cases of leptospirosis in NYC (including our patient).

## Conclusion

In conclusion, leptospirosis has recently come to international attention as a globally important reemerging infectious disease in not only developing countries but in industrialized nations as well. In July 2007, a suspected leptospirosis outbreak was recognized among strawberry harvesters in Germany and was found to be the largest leptospirosis epidemic to occur in Germany since the 1960s [12]. Leptospirosis has also been documented as a militarily relevant infectious disease during times of troop deployment [13]. The implications of this case are noteworthy for several reasons. The case of fulminant leptospirosis presented here should serve to alert health care providers and the general public to the clinical importance of this severe, sometimes fatal, disease. Leptospirosis remains a great burden of infection in third world countries, and mortality remains significant related to lack of a rapid, reliable diagnostic test and the need for a high degree of clinical suspicion. An accurate and quick diagnostic test is warranted in the interest of the individual patient, as well as public health. Recognition of fulminant leptospirosis is especially important because antimicrobial agents can reduce its severity and duration as well as lead to a favorable outcome of this potentially lethal condition.

## Abbreviations

ALP: alkaline phosphatase; ALT: alanine aminotransferase; AST: aspartate aminotransferase; CSF: cerebrospinal fluid; IV: intravenous; MAT: microscopic agglutination test; PCO_2_: partial pressure of carbon dioxide; PO_2_: partial pressure of oxygen.

## Competing interests

The authors declare that they have no competing interests.

## Consent

Written informed consent was obtained from the patient for publication of this case report and accompanying images. A copy of the written consent is available for review by the journal's Editor-in-Chief.

## Authors' contributions

EM was the major contributor to the case presentation, conducted literature review and did manuscript revisions. AK was the major contributor to the discussion section, conducted literature review and did manuscript revisions. EE was involved in direct patient care as the hospitalist. NM was involved in direct patient care as the infectious disease specialist. SE was involved in direct patient care as the renal specialist. All authors have read and approved the final manuscript.
